# The VlMYB149‐VlHIPP30 Regulatory Module Enhances Grapevine Resistance to *Botrytis cinerea* by Activating the Antioxidant System and Copper Metabolism

**DOI:** 10.1111/mpp.70197

**Published:** 2026-01-11

**Authors:** Xiaoli Zhang, Xiangyu Zhou, Guohong Wu, Yanqiu Du, Feng Sun, Songlin Zhou, Yuling Li, Hong Lin, Yong Wang, Changyue Jiang, Yinshan Guo

**Affiliations:** ^1^ College of Horticulture Shenyang Agricultural University Shenyang Liaoning China; ^2^ Research and Development Center for Facility and Specialty Agriculture of Xinjiang Uygur Autonomous Region Turpan China; ^3^ National & Local Joint Engineering Research Center of Northern Horticultural Facilities Design and Application Technology (Liaoning) Shenyang China

**Keywords:** *Botrytis cinerea*, copper metabolism, disease resistance, grapevine, MYB149

## Abstract

Grapevine, as an important economic crop around the world, has generally poor disease resistance in planting. *Vitis vinifera* , in particular, show high susceptibility to grey mould (caused by *Botrytis cinerea*), which leads to a decline in yield and quality. Existing chemical control methods have limitations, including environmental and resistance issues, so breeding disease‐resistant varieties is crucial for sustainable agriculture. In this study, we identified a nuclear membrane‐localised R2R3‐type MYB transcription factor named VlMYB149 whose expression was significantly upregulated following grey mould infection. Overexpression of *VlMYB149* in grapevine and *Arabidopsis* indicated that it significantly enhances resistance to grey mould, characterised by reduced lesion size and inhibited mycelial expansion. *VlMYB149* increased the content of copper and increased the activities of antioxidant enzymes such as catalase (CAT), peroxidase (POD) and superoxide dismutase (SOD). VlMYB149 also directly activated the expression of *VlHIPP30*, which plays a crucial role in the process of copper transport. Overexpression of *VlHIPP30* has been shown to enhance disease resistance by reducing reactive oxygen species (ROS) levels and enhancing copper metabolism. Our findings reveal a novel molecular mechanism model for grapevine resistance to *B. cinerea*, mediated by the synergistic interaction by copper metabolism and the antioxidant system. This study not only provides crucial genetic resources for breeding disease‐resistant crops but also advances our fundamental understanding of plant immunity.

## Graphical abstract

The VlMYB149‐VlHIPP30 module collaboratively facilitates Cu^2+^ accumulation and activates the ROS scavenging pathway to enhance the resistance to grey mould in grapevine.

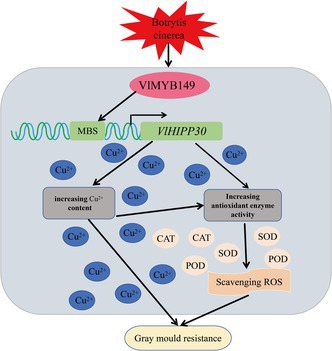

## 1 Introduction

Grapevine is not only rich in nutritional value but also has diverse processing forms (e.g., wine and juice). However, this widely cultivated crop of high economic value is particularly vulnerable to *Botrytis cinerea*, resulting in a decline in fruit yield and quality and consequently causing substantial economic losses (Yue et al. 2015). Grey mould disease ranks among the major diseases plaguing the grape industry. It can result in massive fruit drop, with yield reductions reaching up to 50% (Choquer et al. 2007). *B. cinerea*, a fungal pathogen responsible for grape grey mould disease, exhibits a broad host range, infecting more than 1000 plant species globally (Coertze et al. 2001). The primary entry points for this pathogen are wounds or natural openings in grape leaves, fruits and other plant organs (Martinez et al. 2003). It features complex pathogenic mechanisms, diverse plant metabolites, high levels of cell death, and rapid spread with severe damage (Agüero et al. 2005). Notably, grape germplasms show significant resistance variation, with wild species such as *Vitis labrusca* displaying higher resilience than *Vitis vinifera* (Zhu, Zhang, et al. 2022; Zhu, Zhou, and Zhang 2022). Currently, the primary method for controlling grape grey mould in production is fungicide application, which not only incurs high cultivation costs but also causes environmental pollution (Ma et al. 2021). Understanding the regulatory mechanisms in high‐resistance germplasms is critical for developing high‐quality and disease‐resistant grape varieties.

Copper is an important trace element for normal plant growth and responses to stress, and it is widely involved in various physiological and biochemical reactions in plants as well as plant hormone signal transduction (Aguirre and Pilon 2016; Kumar et al. 2021). It can induce reactive oxygen species (ROS) accumulation through redox reactions, while copper‐binding proteins—primarily antioxidant enzymes—scavenge ROS to maintain copper homeostasis (Pilon 2017; Ravet and Pilon 2013). In plants, copper is transported by the copper transporter (COPT) family, the cation transport heavy metal ATPase P‐type (HMA) family, yellow stripe‐like (YSL) transporter family, and zinc/iron‐regulated transporter‐like protein (ZIP) (Pilon 2017; Ravet and Pilon 2013; Sheng et al. 2021). Copper plays a crucial role in plant disease resistance responses. Previous studies have demonstrated that exogenous application of copper compounds can inhibit the growth of many pathogens (Wang et al. 2015; Yuan et al. 2010). Although Bordeaux mixture, which has copper as a major component, is widely used in agriculture for the control of fungal diseases, the molecular mechanism by which copper signalling regulates plant antiviral defence remains unclear.

Previous studies have shown that, in plants, Heavy Metal‐Associated Isoprenylated Plant Protein (HIPP) functions as a Cu^2+^ metalloprotein, participating in the activity of transporters or metal ‘target proteins’ (Rubino and Franz 2012). The HIPP family is a class of metal‐binding proteins widely found in plants, characterised by the presence of a heavy metal‐associated (HMA) domain and a C‐terminal isoprenylation modification site (CaaX box) (De Abreu‐Neto et al. 2013). The HMA domain within HIPPs is a crucial component in plant defence mechanisms. For instance, studies have shown that rice *OsHIPP19* and *OsHIPP20* are likely susceptibility genes associated with rice blast, because of the fungal effector AVR‐Pik binding to their HMA domains to manipulate their immune‐suppressive functions (Maidment et al. 2021; Oikawa et al. 2024). In contrast, the HMA domain of the rice *RGA5* interacts with the effectors Avr‐Pia and Avr‐CO39, thereby activating the disease resistance function mediated by *RGA4* (Maqbool et al. 2015). Recent research has demonstrated that many HIPP members play key roles in plant responses to biotic stress (Barth et al. 2009; Wang, Li, et al. 2023; Wang, Zhang, et al. 2023; Zheng et al. 2023). *AtHIPP3* in *Arabidopsis* can participate in disease resistance as a regulator via the salicylic acid (SA) pathway (Zschiesche et al. 2015). Additionally, wheat *TaHIPP1* can regulate resistance to powdery mildew through isoprenylation modification, while *AtHIPP26* serves as a host target for the effector AvrPto5 secreted by *Pseudomonas syringae* pv. *actinidiae* (Barth et al. 2009; Wang, Li, et al. 2023; Wang, Zhang, et al. 2023). Although existing studies confirm that the pleiotropic roles of HIPPs in stress resistance, their regulatory networks and mechanisms of functional conservation across species remain to be elucidated. Although a limited number of studies on HIPP have confirmed its role in regulating disease resistance, whether it confers cross‐species resistance in horticultural crops remains unclear. Moreover, the mechanisms underlying HIPP‐mediated plant disease resistance have yet to be fully elucidated.

Plant MYB transcription factors (TFs) are a class of key regulators that play important roles in plant disease resistance (Chezem et al. 2017; Su et al. 2023). MYB TFs can participate in plant defence responses against pathogenic bacteria by modulating the expression of disease resistance‐related genes by binding to specific motifs. Furthermore, they can modulate metabolic pathways, hormone signalling pathways (e.g., SA, jasmonic acid [JA] and ethylene [ET]) and production of ROS, thus inhibiting the invasion of pathogenic bacteria and coordinating the immune system of plant (Jiang et al. 2021; Kishi‐Kaboshi et al. 2018; Seo and Park 2010; Van der Ent et al. 2008; Wang, Li, et al. 2023; Wang, Zhang, et al. 2023). For example, *AtMYB44*, a component of the pathogen‐associated molecular pattern (PAMP)‐triggered immunity (PTI) pathway, confers disease resistance to *Arabidopsis* by promoting the expression of *EIN2*, *MPK3* and *MPK6*. It is also essential for flg22‐induced resistance and ROS generation (Wang, Li, et al. 2023; Wang, Zhang, et al. 2023). Gu et al. (2021) demonstrated that *MdMYB73* in apple up‐regulates the expression of defence‐related genes and induces the synthesis of secondary metabolites, thereby contributing to the defence response against *Botryosphaeria dothidea*. In rose, the *RcMYB84* and *RcMYB123* genes can affect the biosynthesis of phenylpropanoids and flavonoids, and the secondary metabolites produced by these biosynthetic processes can help *Rosa* resist grey mould (Ren et al. 2020). Similarly, in wheat, *TaMYB29* positively regulates defence responses against stripe rust by promoting hydrogen peroxide (H_2_O_2_) accumulation and activating pathogenesis‐related (PR) gene expression (Zhu et al. 2021). In soybean ( *Glycine max* ), *GmMYB33* enhances resistance to *Heterodera glycines* by interacting with downstream defence‐related genes (Lei et al. 2021). In grape, *VqMYB154* from *Vitis quinquangularis* has been shown to positively regulate stilbene synthase genes, promote the accumulation of stilbene phytoalexins and activate the SA pathway, ultimately enhancing disease resistance (Jiang et al. 2021). These findings highlight the specific functions of MYB TFs in disease resistance, wherein their pathogen‐induced expression positions them as critical regulators within plant immune networks. Notably, the role of MYB TFs in the grapevine response to *B. cinerea* remains largely unexplored.

In this study, we integrated RNA‐seq (Su et al. 2023) and reverse transcription‐quantitative PCR (RT‐qPCR) validation to identify a candidate MYB TF, *VlMYB149*, potentially associated with grey mould resistance in grapevines. This study reveals, for the first time, the mechanism that integrates copper metabolism with the antioxidant system to confer resistance against grey mould in grape. The results of this study provide new insights into the mechanism of grape resistance to *B. cinerea*, and provide potential molecular targets and breeding strategies for the cultivation of new grape varieties resistant to *B. cinerea*.

## 2 Results

### 2.1 The Negative Effect of Exogenous Cu^2+^ Treatment on the Growth of *B. cinerea*

**FIGURE 1:**
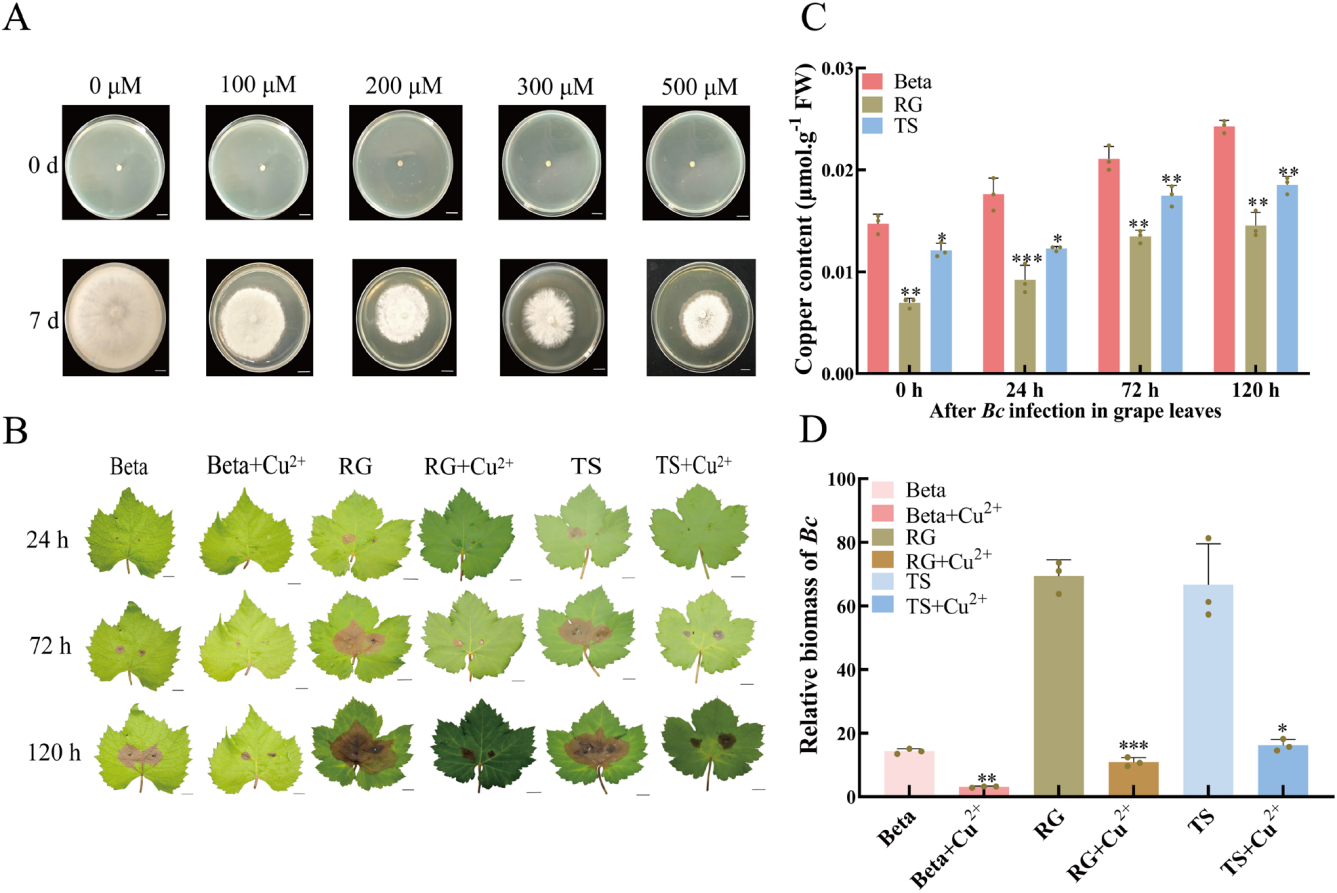
The effect of exogenous Cu^2+^ treatment on the growth of *Botrytis cinerea*. (A) Observational experiment on the growth morphology and expansion of *B. cinerea* under different concentrations of Cu^2+^ treatment over 7 days. (B) Phenotypic observation experiment of grape leaves of three varieties after *B. cinerea* infection under Cu^2+^ treatment or without Cu^2+^ treatment. RG, Red Globe; TS, Thompson Seedless. (C) Dynamic changes in copper content at different time points after *B. cinerea* infection in three grape varieties. (D) Determination of *B. cinerea* biomass in grape leaves of three varieties at 120 h after infection, with or without Cu^2+^ treatment. The values are means ± SE from three independent replicates. Significant differences were defined by Student's *t* test and ANOVA (*p < 0.05, **p < 0.01, ***p < 0.001).

To investigate the effect of Cu^2+^ on the growth of *B. cinerea*, we used the agar plate method, inoculating mycelium with identical growth periods and states onto potato dextrose agar (PDA) plates containing different concentrations (0, 100, 200, 300 and 500 μM) of Cu^2+^. The results showed that Cu^2+^ treatment at different concentrations effectively inhibited the growth and spread of *B. cinerea* (Figure 1A). The higher the Cu^2+^ concentration, the stronger the inhibitory effect on *B. cinerea*, as evidenced by the slower growth rate and the smaller the colony diameter.

To further clarify the role of Cu^2+^ in grape resistance to *B. cinerea*, we inoculated the pathogen on leaves from three grape varieties—Thompson Seedless (TS), Red Globe (RG) and Beta—that were either pretreated with Cu^2+^ or left untreated. The results showed that exogenous Cu^2+^ treatment inhibited the growth and spread of *B. cinerea* in grape leaves. Specifically, 24 h after inoculation, the control group of grape leaves that had not undergone Cu^2+^ treatment exhibited browning and rotting at the inoculation site, with fungal hyphae growing and spreading. In contrast, the group of grape leaves treated with Cu^2+^ showed no obvious signs of rot at the inoculation site, and the growth and spread of the pathogen were significantly inhibited (Figure 1B). Copper content measurements were conducted on grapevine leaves at various stages. Copper content fluctuated over time in all varieties following *B. cinerea* infection. Beta exhibited significantly higher copper accumulation than TS and RG at all stages (Figure 1C). Additionally, the biomass of *B. cinerea* in Cu^2+^‐treated grape leaves was approximately 80% lower than that in untreated leaves (Figure 1D). These results indicate that Cu^2+^ treatment enhances grapevine resistance to *B. cinerea*.

### 2.2 Screening and Basic Characteristic Analysis of *MYB149*

**FIGURE 2:**
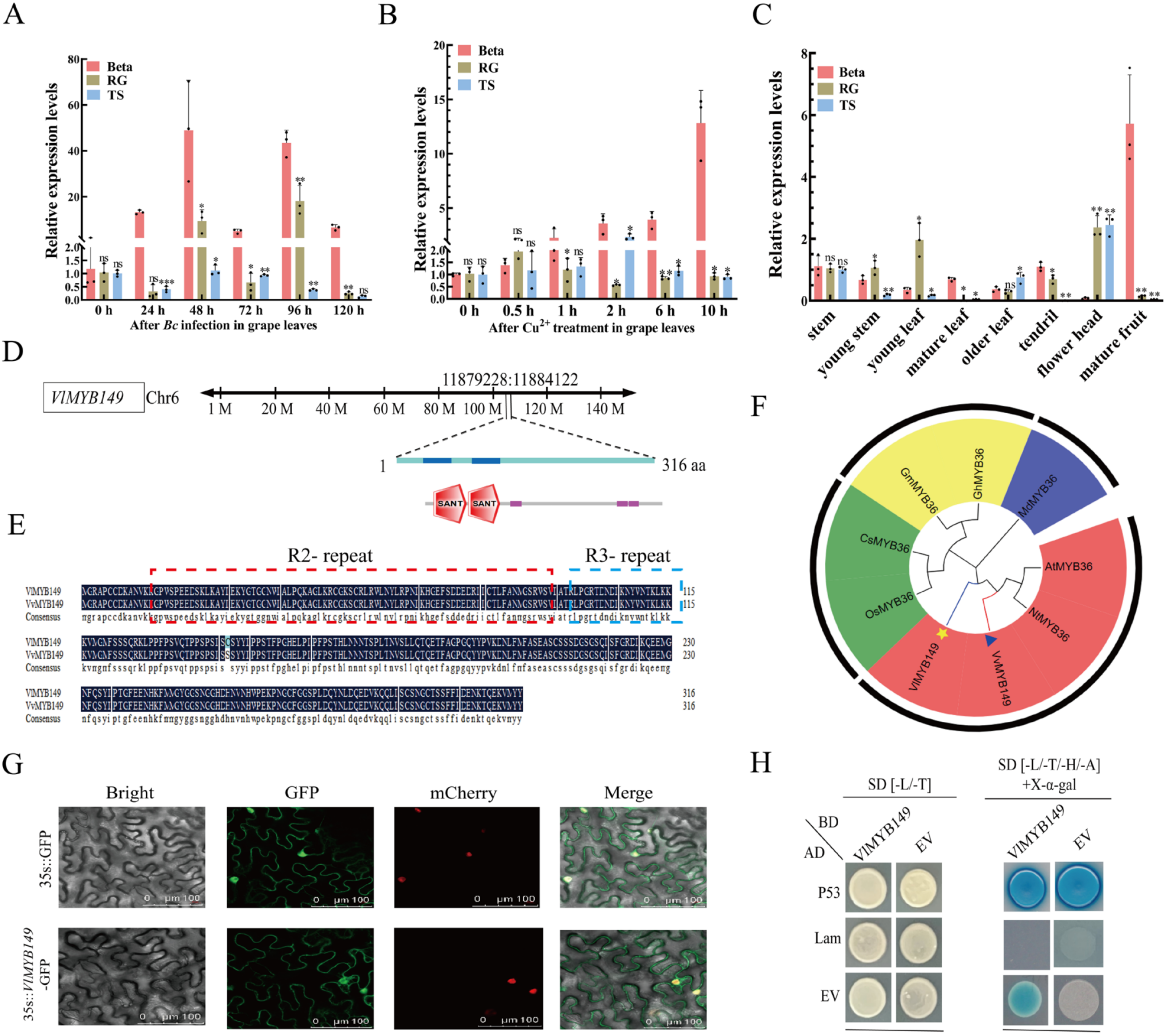
Analysis of the expression patterns under *Botrytis cinerea* induction in grapevines and basic characteristics of *MYB149*. (A) Analysis of the expression of *MYB149* at different stages under *B. cinerea* infection in three varieties. RG, Red Globe; TS, Thompson Seedless. (B) Relative expression levels of *MYB149* at different time points in grape leaves of three varieties after Cu^2+^ treatment. (C) Differential expression analysis of *MYB149* in different tissues of three varieties. (D) Chromosomal localisation of *MYB149*. (E) Amino acid sequences of MYB149 in *Vitis labrusca* ‘Beta’ and *Vitis vinifera* ‘Thompson Seedless’. (F) Analysis of the phylogenetic tree of MYB149. (G) Subcellular localisation of *VlMYB149* in *Nicotiana benthamiana* leaves. (H) Yeast self‐activation analysis of VlMYB149. The values are means ± SE from three independent replicates. Significant differences were defined by Student's *t* test and ANOVA (*p < 0.05, **p < 0.01, ***p < 0.001).

Through RNA‐seq analysis of Beta and RG subjected to varying durations of *B. cinerea* infection, it was observed that 32 MYB genes were upregulated in Beta, while 50 MYB genes were upregulated in RG. Among these, 22 MYB genes were upregulated in both cultivars. Eight MYB genes (*MYB1*, *MYB38*, *MYB53*, *MYB57*, *MYB68*, *MYB82* and *MYB149*) exhibited specific upregulation in Beta, with no corresponding upregulation detected in RG (Figure S1). Subsequent RT‐qPCR analysis of these eight MYB genes revealed that *MYB149* displayed consistently high expression levels at 24, 48, 72, 96 and 120 h post‐inoculation (hpi), compared to the other seven MYB genes (Figure S2, Figure 2A). Notably, Beta exhibited a marked induction of *MYB149* expression under *B. cinerea* infection, which was maintained at high levels, particularly at 24–48 hpi, suggesting a strong transcriptional activation. In contrast, a gradual upregulation was observed in RG during the later phase of infection (48–96 hpi), though the overall induction was subdued compared to that in Beta. TS displayed only marginal fluctuation in *MYB149* transcript levels throughout the infection period, consistent with a weak transcriptional response. These findings highlight *MYB149* as a promising candidate gene for further investigation into disease resistance mechanisms. In addition, we also found that the expression level of *MYB149* was upregulated by Cu^2+^ treatment. At 1 h of Cu^2+^ treatment, the expression level of the *MYB149* was approximately twice as high as that of the control (0 h), and with the extension of treatment time, the expression level of the *MYB149* continued to increase (as shown in Figure 2B). Based on the above results, we speculate that *MYB149* may play a regulatory role in the copper ion‐mediated grape disease resistance pathway.

The results of tissue‐specific expression analysis indicate that *MYB149* exhibited higher expression levels in leaves, with the highest levels detected in mature berries, followed by young and old leaves. Among non‐foliar tissues, flower head and fruits showed relatively strong expression, whereas stems and tendrils displayed lower transcript abundance (Figure 2C). Notably, *MYB149* expression in tendrils, mature leaves and berries of Beta was significantly higher compared to the susceptible cultivars RG and TS (Figure 2C). Located on chromosome 6 (coordinates 11,879,228–11,884,122), *MYB149* encodes a 316‐amino‐acid protein with two SANT domains (R2R3 repeats)‐the DNA‐binding core motif characteristic of R2R3‐MYB TFs (Figure 2D). Similarly, a comparison of the amino acid sequences between VlMYB149 from *Vitis labrusca* ‘Beta’ and VvMYB149 from *V. vinifera* ‘Thompson Seedless’ revealed that they differ by only a single amino acid residue (Figure 2E). Phylogenetic tree analysis indicates that VlMYB149 and VvMYB149 share high similarity with AtMYB36 in *Arabidopsis* and *Nicotiana tabacum* NtMYB149 (Figure 2F). Moreover, VlMYB149 showed nuclear membrane localisation in *Nicotiana bethamiana* leaves (Figure 2G). Yeast self‐activation assays indicated that VlMYB149 exhibits transcriptional activation activity in yeast (Figure 2H).

### 2.3 Disease Resistance Functional Assay of OE‐ *VlMYB149* and RNAi‐ *VlMYB149* in Grape Leaves and Berries

**FIGURE 3:**
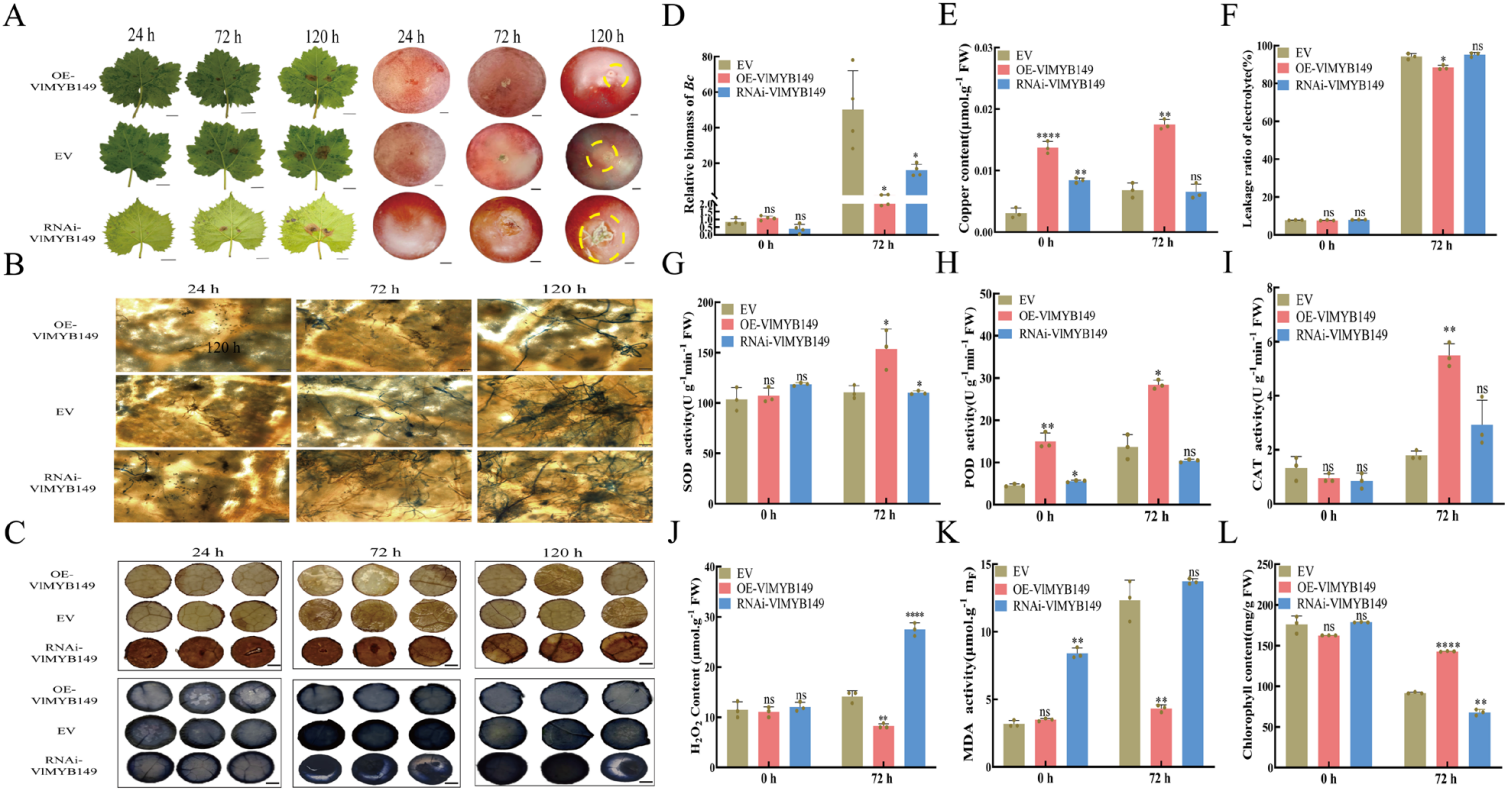
VlMYB149 enhances grapevine resistance to *Botrytis cinerea* by enhancing the antioxidant system and copper metabolism. (A) Effects of transient overexpression (OE) and silencing (RNAi) of *VlMYB149* on *B. cinerea* resistance of grape. EV, empty vector. (B) Trypan blue staining to detect the development of *B. cinerea.* (C) 3,3′‐diaminobenzidine (upper panels) and nitroblue tetrazolium (lower panels) staining after inoculation with *B. cinerea.* (D) Analysis of relative biomass in leaves after inoculation with *B. cinerea.* (E) Analysis of Cu^2+^ content in leaves after inoculation with *B. cinerea.* (F) Analysis of electrolyte leakage in leaves after inoculation with *B. cinerea.* (G) Analysis of superoxide dismutase (SOD) content in leaves after inoculation of *B. cinerea.* (H) Analysis of peroxidase (POD) content in leaves after inoculation of *B. cinerea.* (I) Analysis of catalase (CAT) content in leaves after inoculation of *B. cinerea.* (J) Analysis of H_2_O_2_ content in leaves after inoculation of *B. cinerea.* (K) Analysis of malondialdehyde (MDA) content in leaves after inoculation of *B. cinerea.* (L) Analysis of chlorophyll content in leaves after inoculation with *B. cinerea*. The values are means ± SE from three independent replicates. Significant differences were defined by Student's *t* test and ANOVA (*p < 0.05, **p < 0.01, ****p < 0.0001).

To elucidate the effect of *VlMYB149* on grapevine resistance to *B. cinerea*, we obtained *VlMYB149* transgenic leaves and berries. And after inoculation with *B. cinerea*, the lesion area was significantly smaller on OE‐*VlMYB149* leaves compared to empty vector (EV) and RNAi‐*VlMYB149* controls (Figure 3A). Similar phenotypic results were also observed in RG fruits (Figure 3A). Histochemical staining showed that 24 hpi, abundant fungal hyphae were visible in tissue sections of control leaves, whereas only sparse hyphal distribution was detected in OE‐*VlMYB149* leaves. By 72 hpi, quantitative analysis indicated significantly lower hyphal density in overexpressing leaves, suggesting that *VlMYB149* effectively inhibits hyphal expansion (Figure 3B).

The *B. cinerea* colonisation assays revealed no significant difference in fungal biomass between OE‐*VlMYB149* and control plants under normal conditions. However, under infection, OE‐*VlMYB149* plants exhibited significantly reduced fungal colonisation, consistent with their enhanced disease resistance (Figure 3D). Copper content analysis showed that *VlMYB149* significantly increased copper levels in plant tissues, with *B. cinerea* infection further elevating copper accumulation (Figure 3E). Additionally, *VlMYB149*‐overexpressing grape leaves lost less chlorophyll compared to control and RNAi leaves under infection conditions (Figure 3L). As illustrated in Figure 3G–I, the results of physiological parameter measurements demonstrated that under *B. cinerea* infection, the contents of antioxidant enzymes such as superoxide dismutase (SOD), peroxidase (POD) and catalase (CAT) were significantly elevated in OE‐*VlMYB149* samples, enhancing their ability to scavenge ROS and resulting in reduced ROS accumulation compared to EV and RNAi‐*VlMYB149* controls. Electrolyte leakage (Figure 3F) and malondialdehyde (MDA) content (Figure 3K) of OE‐*VlMYB149* leaves were significantly reduced, which collectively indicates that the plants' disease resistance was enhanced.

To further validate ROS accumulation, transiently transformed TS leaves were sampled at 0, 24 and 72 hpi and stained with nitroblue tetrazolium (NBT) and 3,3′‐diaminobenzidine (DAB) (H_2_O_2_ oxidises DAB to form brown precipitates, while O^2−^oxidises NBT to form blue precipitates). Results showed lighter staining (pale dark brown and dark blue) in *VlMYB149*‐overexpressing leaves compared to control leaves and RNAi‐*VlMYB149* leaves, indicating reduced ROS accumulation (Figure 3C). H_2_O_2_ content measurements in Figure 3J aligned with the staining results. Collectively, these results suggest that *VlMYB149* may mediate its specific disease resistance function by regulating the activity of antioxidant enzymes (CAT, SOD, POD) and intracellular copper content.

### 2.4 Disease Resistance Functional Assay of *VlMYB149* in *Arabidopsis*

**FIGURE 4:**
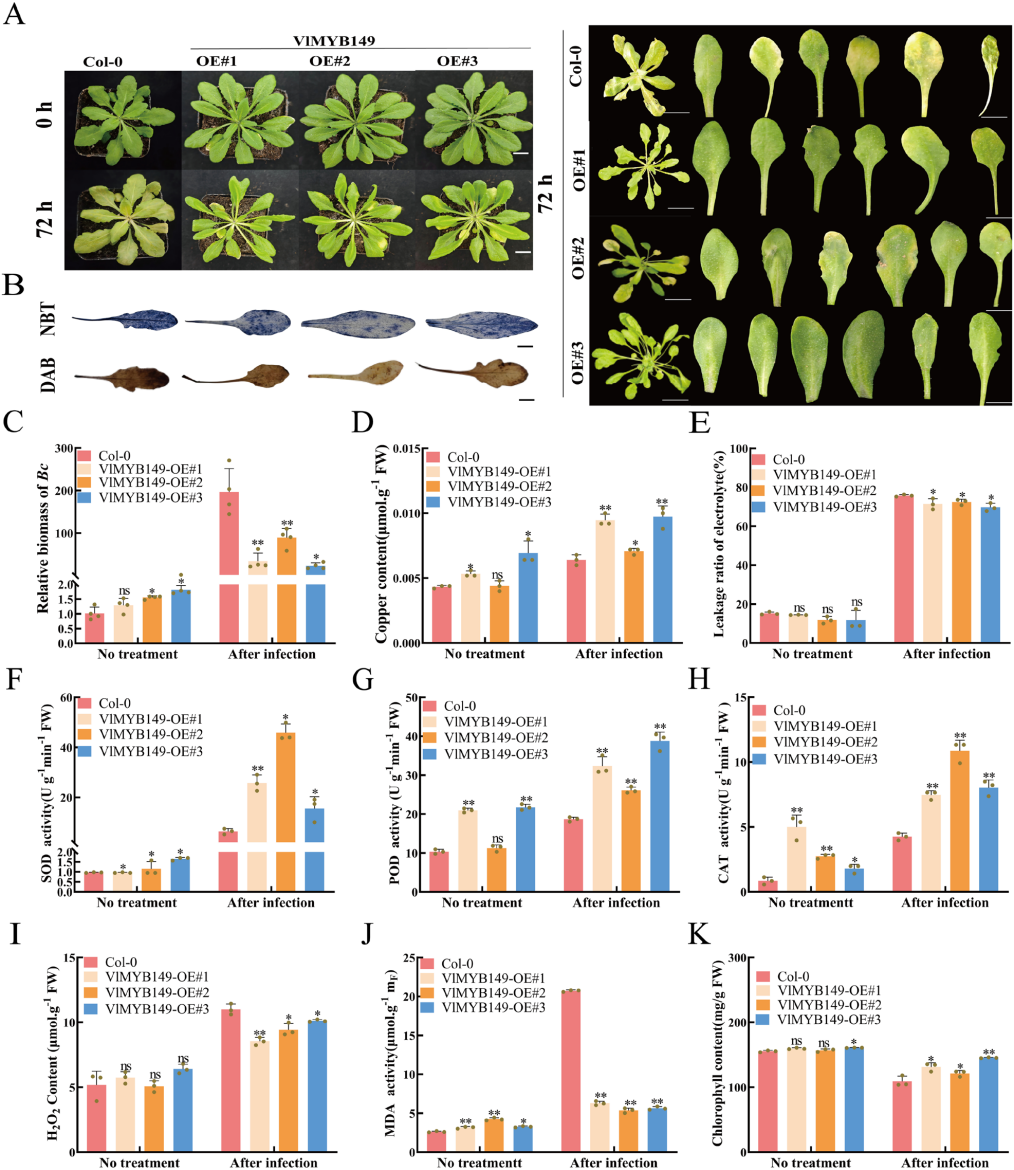
Identification of disease resistance related function and analysis of physiological indicators of *VlMYB149*‐overexpressing *Arabidopsis thaliana* . (A) Effects of stable overexpression *VlMYB149* on *Botrytis cinerea* resistance of *A. thaliana* . (B) Nitroblue tetrazolium (NBT) and 3,3′‐diaminobenzidine (DAB) staining after inoculation with *B. cinerea.* (C) Analysis of relative biomass in leaves after inoculation with *B. cinerea.* (D) Analysis of Cu^2+^ content in leaves after inoculation with *B. cinerea.* (E) Analysis of electrolyte leakage in leaves after inoculation with *B. cinerea.* (F) Analysis of superoxide dismutase (SOD) content in leaves after inoculation of *B. cinerea.* (G) Analysis of peroxidase (POD) content in leaves after inoculation of *B. cinerea.* (H) Analysis of catalase (CAT) content in leaves after inoculation of *B. cinerea.* (I) Analysis of H_2_O_2_ content in leaves after inoculation of *B. cinerea.* (J) Analysis of malondialdehyde (MDA) content in leaves after inoculation of *B. cinerea.* (K) Analysis of chlorophyll content in leaves after inoculation with *B. cinerea*. The values are means ± SE from three independent replicates. Significant differences were defined by Student's *t* test and ANOVA (*p < 0.05, **p < 0.01).

To validate the disease resistance regulatory function of *VlMYB149* across species, *Arabidopsis* overexpressing lines (OE#1, OE#2 and OE#3) were selected for *B. cinerea* infection treatment (Figure 4A). Under normal control conditions, both EV and OE‐*VlMYB149* plants grew normally. However, under *B. cinerea* infection stress, *OE‐VlMYB149* plants exhibited healthier leaf appearance, and lower leaf wilting and disease symptoms compared to EV controls (Figure 4A). *Arabidopsis* leaves were sampled at 72 hpi and stained with NBT and DAB to detect ROS accumulation. DAB and NBT staining showed darker colouration in EV controls compared to OE‐*VlMYB149* leaves, indicating higher ROS accumulation in EV plants under *B. cinerea* infection (Figure 4B). Quantitative analysis of fungal biomass revealed no significant difference in *B. cinerea* colonisation between OE‐*VlMYB149* and EV plants under normal conditions. However, OE‐*VlMYB149* plants exhibited significantly reduced fungal colonisation, consistent with the disease resistance phenotype observed in OE‐*VlMYB149* grapevine materials (Figure 4C). Copper content analysis showed that OE‐*VlMYB149* significantly increased copper levels in plant tissues, with *B. cinerea* infection further enhancing copper accumulation (Figure 4D). Additionally, overexpression of *VlMYB149* significantly decreased electrolyte leakage and MDA content of leaves, indicating enhanced disease resistance (Figure 4E,J). Measurements of CAT, POD, SOD and H_2_O_2_ contents revealed that under *B. cinerea* infection, OE‐*VlMYB149* plants produced higher levels of antioxidant enzymes, enhancing their ability to scavenge ROS and resulting in reduced ROS accumulation compared to EV plants (Figure 4F–I). Furthermore, overexpressing *Arabidopsis* plant lost less chlorophyll compared to control plants under infection conditions (Figure 4K). In summary, overexpression of *VlMYB149* also enhanced the disease resistance of the model plant *Arabidopsis* through increasing copper content and antioxidant enzyme activity.

### 2.5 *VlMYB149* Directly Activates *VlHIPP30* Expression in Grapevine

**FIGURE 5:**
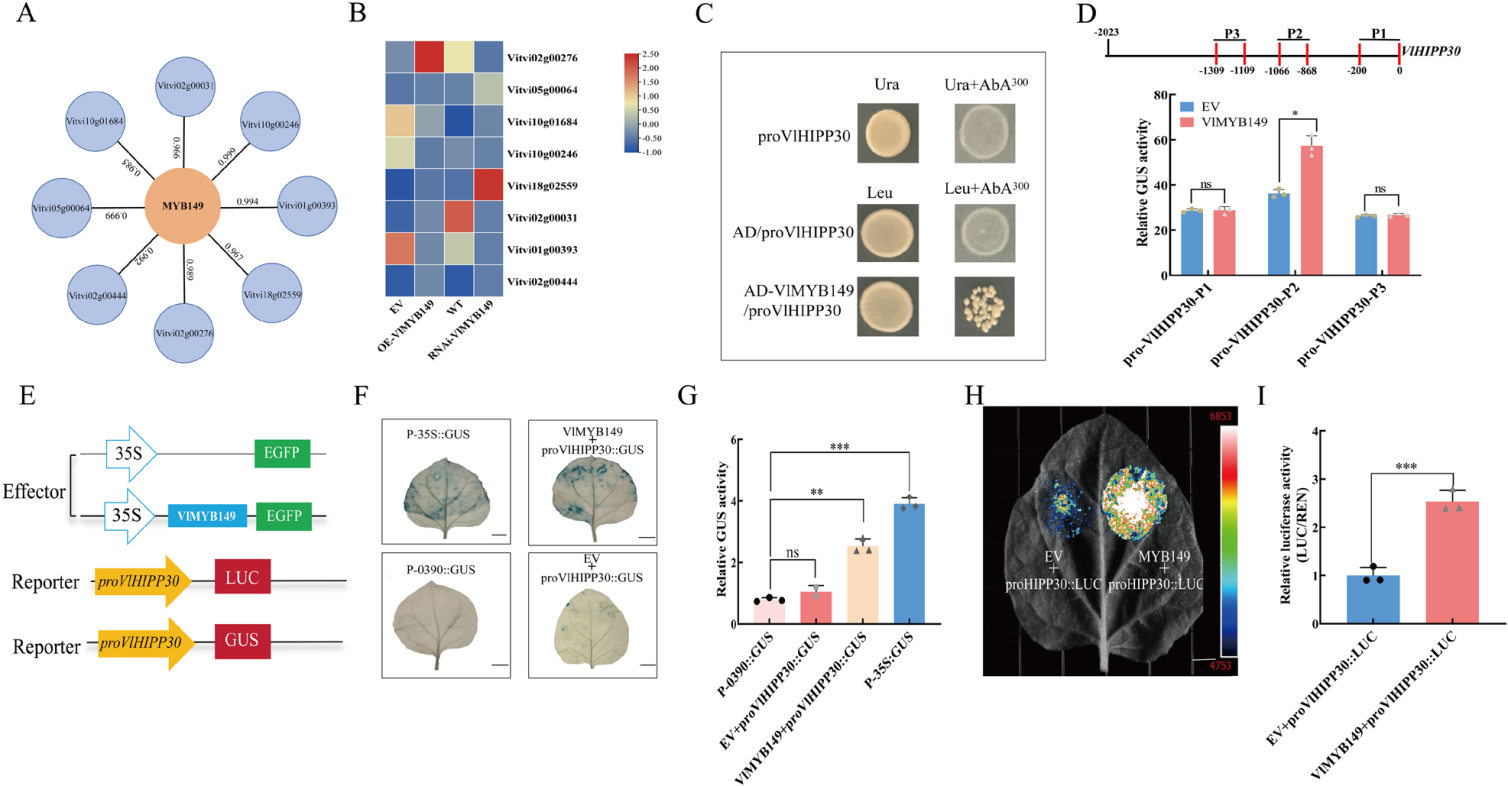
VlMYB149 positively regulates the expression of *VlHIPP30* by directly activating its promoter. (A) Candidate genes co‐expressed with *MYB149* screened by RNA‐seq. (B) Relative expression levels of candidate genes over time in overexpression (OE) and silenced (RNAi) lines after *Botrytis cinerea* infection. WT, wild type; EV, empty vector. (C) Verification of VlMYB149 binding to the promoter of *VlHIPP30* by yeast one‐hybrid assay. (D) β‐glucuronidase (GUS) reporter assay was used to detect the interaction between VlMYB149 and *VlHIPP30* promoter fragments (P1, P2, P3) in *Nicotiana benthamiana* leaves. (E) Construction of GUS vector and luciferase (LUC) vector. (F) GUS reporter assay was used to detect the interaction between VlMYB149 and *VlHIPP30* promoter in *N. benthamiana* leaves. (G) Measurement of GUS activity. (H) LUC reporter assay was used to detect the interaction between VlMYB149 and *VlHIPP30* promoter in *N. benthamiana* leaves. (I) Measurement of LUC activity. The values are means ± SE from three independent replicates. Significant differences were defined by ANOVA (*p < 0.05, **p < 0.01, ***p < 0.001).

Based on previous research findings, copper exhibits disease‐resistant properties, and exogenous copper application can induce the expression of *VlMYB149*. Therefore, we speculated that *VlMYB149* may participate in regulating the expression of genes associated with copper metabolism pathways. To elucidate the molecular mechanism underlying *VlMYB149*‐mediated resistance to grey mould in grapevine, we identified eight potential genes co‐expressed with *VlMYB149* through transcriptome analysis of grape leaves collected at various time points after *B. cinerea* infection. Then we conducted RT‐qPCR analysis of these candidate genes in grape leaves overexpressing *VlMYB149* (Figure 5A,B). Among them, *HIPP30* emerged as a key candidate gene, as it displayed higher expression levels in OE‐*VlMYB149* leaves compared to the other genes.

To verify whether VlMYB149 directly binds to the promoter of *VlHIPP30*, a 2 kb promoter sequence of *VlHIPP30* was cloned from Beta leaves. Then, the promoter sequence of *VlHIPP30* and coding sequence of *VlMYB149* were inserted into the pAbAi vector and the pGADT7 (AD) vector, respectively (Figure 5E). Yeast one‐hybrid (Y1H) assays revealed that VlMYB149 bound to the *VlHIPP30* promoter (Figure 5C). To further clarify the regulatory effect of VlMYB149 on *VlHIPP30*, the *VlHIPP30* promoter was inserted into luciferase (LUC) and β‐gluuronidase (GUS) reporter vectors and assays were conducted in *Nicotiana benthamiana* leaves. Results showed that VlMYB149 significantly enhanced the LUC activity driven by *VlHIPP30* promoter compared to empty vector (EV) control (Figure 5H,I), demonstrating that VlMYB149 activated the *VlHIPP30* promoter. GUS assays showed consistent results (Figure 5F,G).

To identify the binding region of VlMYB149 on the *VlHIPP30* promoter, we selected three different fragments (P1, P2 and P3) from the *VlHIPP30* promoter region based on the distribution of MYB binding sites (MBSs). The P1 and P3 fragments contained a SAGGSATAKA motif; the P2 fragment contained a TAGTTGTACAGG motif. These fragments were individually inserted into a GUS vector (pC0390‐35S‐GUS) as reporter, and *VlMYB149* was inserted into the pCAMBIA2300 vector as effector. These reporters and effector were co‐transformed into *N. benthamiana* leaves. The result showed in Figure 5D that VlMYB149 markedly enhanced GUS activity only when driven by the P2 fragment of *VlHIPP30* promoter compared to EV controls. These findings indicate that VlMYB149 directly binds to the TAGTTGTACAGG motif in the P2 fragment of the *VlHIPP30* promoter and activates expression of this gene in grapevine.

### 2.6 Screening and Basic Characteristic Analysis of *HIPP30*

**FIGURE 6:**
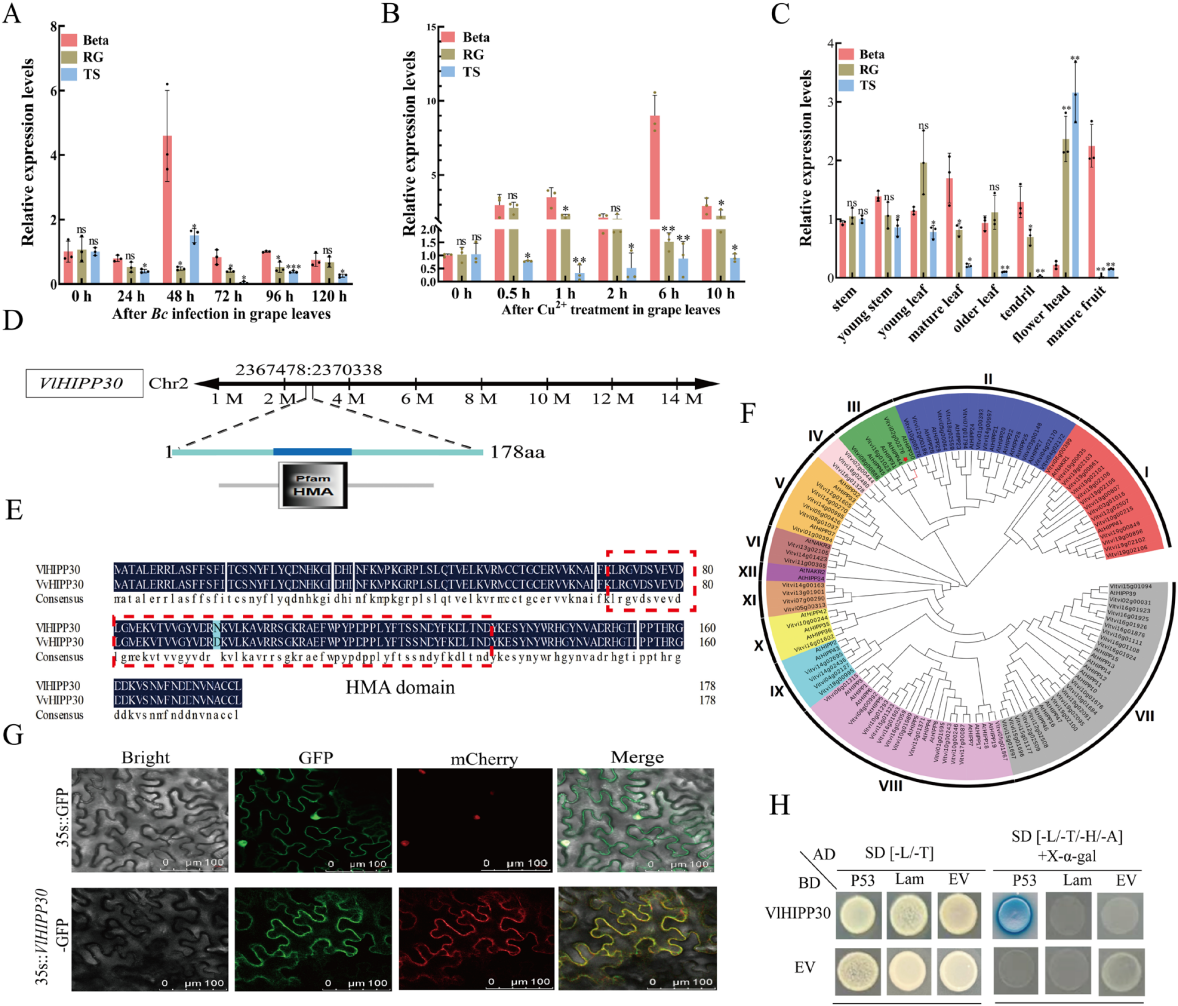
Analysis of the localisation, expression pattern, and transcriptional activation function of *HIPP30*. (A) Analysis of the expression of *HIPP30* at different stages under *Botrytis cinerea* infection in three varieties. RG, Red Globe; TS, Thompson Seedless. (B) The expression levels of *HIPP30* at different time points in grape leaves of three varieties after Cu^2+^ treatment. (C) Expression analysis of *HIPP30* in different tissues of three varieties. (D) Chromosomal localisation of *VlHIPP30*. (E) Amino acid sequences of HIPP30 in *Vitis labrusca* ‘Beta’ and *Vitis vinifera* ‘Thompson Seedless’. (F) Analysis of the phylogenetic tree of HIPP30. (G) Subcellular localisation of VlHIPP30. (H) Yeast self‐activation analysis of VlHIPP30. The values are means ± SE from three independent replicates. Significant differences were defined by Student's *t* test and ANOVA (*p < 0.05, **p < 0.01).

To further investigate the expression pattern of the *HIPP30* gene with *B. cinerea* infection in resistant or susceptible grapevine, we also monitored the expression levels of *HIPP30* in TS, Beta and RG grape leaves at 0, 24, 48, 72, 96 and 120 hpi. As shown in Figure 6A, following inoculation, the expression levels of *HIPP30* exhibited a trend of initially increasing and then decreasing over time. At 48 hpi, the expression levels of *HIPP30* reached a peak, with the expression being induced by more than fourfold compared to the control (0 hpi). Additionally, we found that *HIPP30* expression were also upregulated by Cu^2+^ treatment. At 0.5 h after Cu^2+^ treatment, the *HIPP30* expression levels were approximately twice that of the control (Figure 6B). Notably, the three grape cultivars exhibited distinct transcriptional responses to Cu^2+^ treatment. Beta displayed rapid and significant upregulation of *VlHIPP30* following Cu^2+^ exposure, with the expression level peaking at 6 h, which indicates a robust transcriptional activation. RG showed intermediate expression patterns, where its *Vv* *HIPP30* expression level was consistently higher than in TS but significantly lower than *VlHIPP30* in Beta across all treatment time points. In contrast, TS exhibited minimal transcriptional induction throughout the entire Cu^2+^ treatment period, suggesting a weak responsive capacity to Cu^2+^ stimulation. Moreover, the tissue‐specific expression pattern assay of *HIPP30* in grapevines showed that *HIPP30* was detected in all tissues. Except for young leaves and flower heads, the expression level of *HIPP30* in various tissues of Beta was higher than that in TS and RG; Additionally, the expression level was higher in the mature fruits and mature leaves of Beta, followed by tendrils and older leaves, and the lowest expression level was observed in flower heads (Figure 6C).

We cloned the coding sequences of *VvHIPP30* and *VlHIPP30* from TS and Beta, respectively. *HIPP30* is located on grape chromosome 2 (interval 2367478–2,370338), encoding 179 amino acids and containing 1 HMA domain (Figure 6D). Meanwhile, the amino acid sequences of VlHIPP30 and VvHIPP30 share 99% similarity, differing by only a single amino acid (Figure 6E). Phylogenetic tree analysis showed that VlHIPP30 belongs to the HIPP family III subfamily, which is most closely related to AtHIPP30 and AtHIPP44 in *Arabidopsis* (Figure 6F). Subcellular localisation analysis showed that VlHIPP30 was localised on the cell membrane (Figure 6G). To investigate the transcriptional activity of VlHIPP30, the coding sequence of *VlHIPP30* was inserted into pGBKT7 (BD) vector, forming *BD‐VlHIPP30*. Then, the EV and *BD‐VlHIPP30* were transformed into yeast strain Y2H. The results showed that VlHIPP30 had no transcriptional activation activity in yeast (Figure 6H).

### 2.7 Disease Resistance Functional Analysis of *VlHIPP30* in Grape Leaves and Berries

**FIGURE 7:**
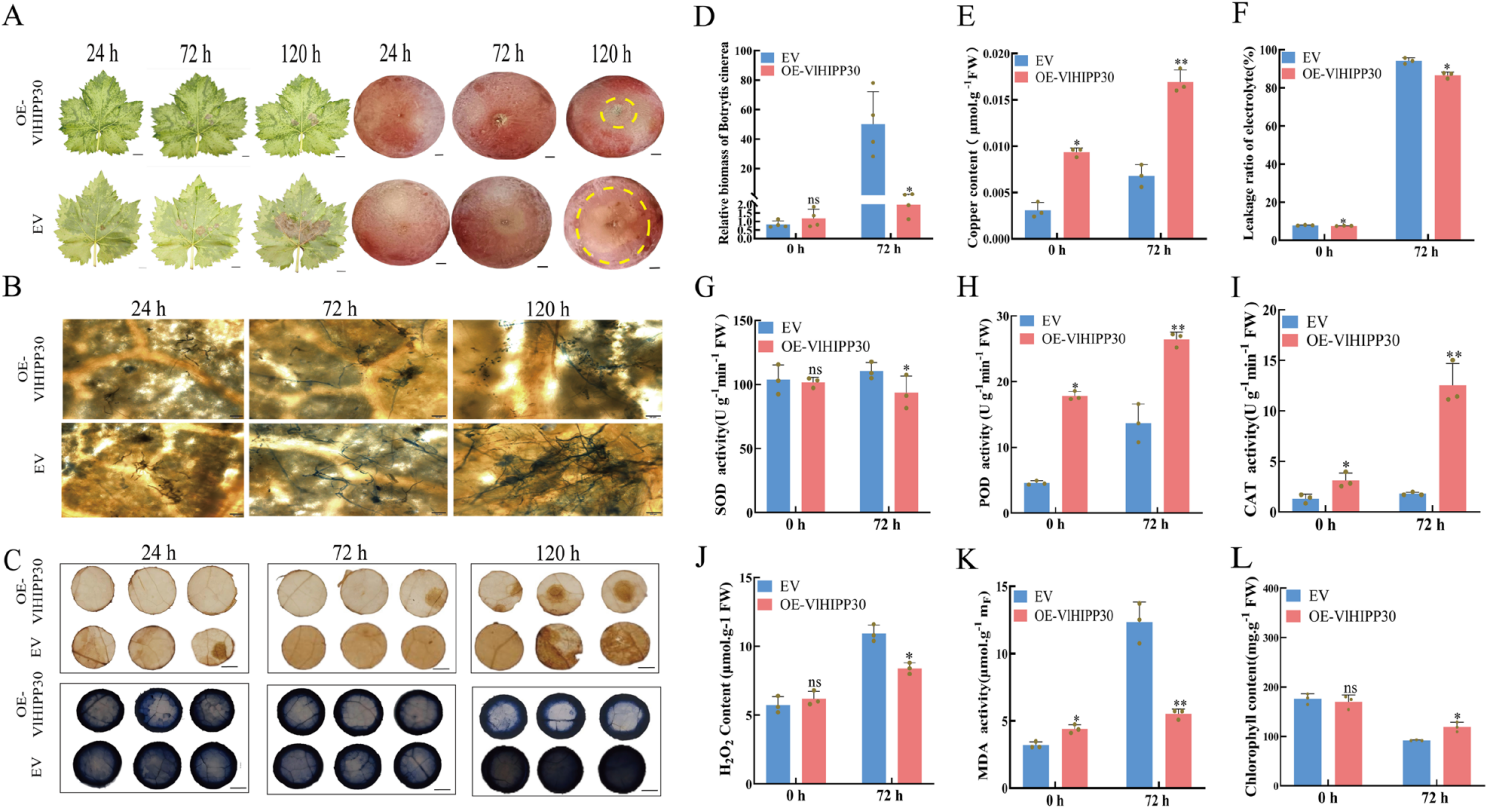
The overexpression of *VlHIPP30* in grapevine enhances the resistance to *Botrytis cinerea* by promoting antioxidant ability and copper metabolism. (A) Effects of transient overexpression of *VlHIPP30* on *B. cinerea* resistance of grape. (B) Trypan blue staining to detect the development of *B. cinerea.* Scale bar = 100 μm. (C) 3,3′‐diaminobenzidine (upper panels) and nitroblue tetrazolium (lower panels) staining after inoculation with *B. cinerea.* (D) Analysis of relative biomass in leaves after inoculation with *B. cinerea.* (E) Analysis of Cu^2+^ content in leaves after inoculation with *B. cinerea.* (F) Analysis of electrolyte leakage in leaves after inoculation with *B. cinerea.* (G) Analysis of superoxide dismutase (SOD) content in leaves after inoculation of *B. cinerea.* (H) Analysis of peroxidase (POD) content in leaves after inoculation of *B. cinerea.* (I) Analysis of catalase (CAT) content in leaves after inoculation of *B. cinerea.* (J) Analysis of H_2_O_2_ content in leaves after inoculation of *B. cinerea.* (K) Analysis of malondialdehyde (MDA) content in leaves after inoculation of *B. cinerea.* (L) Analysis of chlorophyll content in leaves after inoculation with *B. cinerea*. The values are means ± SE from three independent replicates. Significant differences were defined by Student's *t* test and ANOVA (*p < 0.05, **p < 0.01).

To verify the *B. cinerea*‐resistant function of *VlHIPP30*, we performed transient overexpression in leaves of TS and fruits of RG. The coding sequence of *VlHIPP30* was inserted into the pCAMBIA‐2300 vector to construct OE‐*VlHIPP30*. OE‐*VlHIPP30* and EV were transformed into leaves of TS via *Agrobacterium* *tumefaciens* GV3101. RT‐qPCR results showed that *VlHIPP30* expression was significantly upregulated in OE‐*VlHIPP30* leaves compared with EV. Subsequently, EV and OE‐*VlHIPP30* leaves were subjected to *B. cinerea* infection treatment: 24 hpi, EV leaves showed obvious lesion necrosis while OE‐*VlHIPP30* leaves remained normal; 72 hpi, the necrotic area of EV leaves continued to expand and the biomass of *B. cinerea* was significantly higher than that of the EV; this phenotypic difference also existed in RG fruits (Figure 7A,D). As illustrated in Figure 7B, more fungal hyphae were produced in the empty leaves. Additionally, DAB and NBT staining assays revealed higher ROS accumulation in EV leaves compared with OE‐*VlHIPP30* leaves (Figure 7C). Similarly, the copper content in the leaves of OE‐*VlHIPP30* also increased after *B. cinerea* infection (Figure 7E). In addition, under *B. cinerea* infection, the antioxidant enzyme activities (CAT, POD, SOD) in OE‐*VlHIPP30* leaves were significantly higher than those in EV controls (Figure 7G–I); correspondingly, the relative electrolyte leakage, H_2_O_2_ and MDA contents in the OE‐*VlHIPP30* group were significantly lower than those in EV controls (Figure 7F,J,K). Furthermore, under *B. cinerea* infection, the chlorophyll content loss was significantly lower in OE‐*VlHIPP30* grape leaves than in the control (Figure 3L). The above results indicate that the expression of *VlHIPP30* boosts the defence of grapevine against *B. cinerea*.

## 3 Discussion

Past investigations have indicated that HIPP proteins contribute to disease resistance through their family‐specific HMA domains or C‐terminal isoprenylation sites (De Abreu‐Neto et al. 2013). The plasma membrane (PM) plays a crucial role in regulating the reception and transmission of extracellular signals during plant immune responses. For instance, in *Arabidopsis*, flagellin induces the association of FLS2 with *BAK1*, recruiting cytoplasmic signalling components such as *BIK1* and *Pub12/13* to fine‐tune the activation of the pattern recognition receptor (PRR) complex (Gu et al. 2021; Lu et al. 2011; Lu et al. 2010). Moreover, Wang, Li, et al. (2023) and Wang, Zhang, et al. (2023) demonstrated that the PM‐anchored HIPP1‐V/CMPG1‐V complex perceives *Blumeria graminis* f. sp. *tritici* infection, thereby initiating resistance. These findings underscore the importance of rapid and efficient defence mechanisms at the PM for successful plant immunity. Notably, *VlHIPP30* is localised to the cell membrane, suggesting its potential involvement in signal transduction and copper transport. Previous studies have shown that MYB TFs typically promote disease resistance through hormone pathways. For example, apple *MYB73* enhances resistance by elevating SA levels and upregulating SA‐related synthesis and signalling genes (Gu et al. 2021), while ET‐induced tobacco *MYB4L* interacts with downstream regulators to form a feedforward loop that strengthens antiviral resistance (Zhu, Zhang, et al. 2022; Zhu, Zhou, and Zhang 2022). In contrast, our study revealed that VlMYB149 exhibits localisation in both the cell membrane and the nucleus (Figure 2F), diverging from the conventional nuclear localisation of most TFs. Given that VlHIPP30 is exclusively membrane‐localised and contains an HMA domain known for metal ion binding, we propose that VlMYB149 may facilitate copper accumulation via interaction with VlHIPP30 at the cell membrane. This mechanism could contribute to the rapid establishment of an effective PM‐based defence system against pathogen invasion.

ROS play a complex dual role in plant disease resistance: moderate production serves as key signalling molecules to activate immune responses; however, excessive accumulation induces oxidative damage, suppresses defences, and promotes disease progression (Apel and Hirt 2004). This dual effect depends on the delicate balance between ROS production and removal (Bailey‐Serres and Mittler 2006). Notably, cell death induced by excessive ROS accumulation paradoxically benefits saprophytic pathogens like *B. cinerea*, which rely on nutrients from dead host tissues. Thus, the success of plant disease resistance largely hinges on the precise regulation of intracellular ROS levels. In this study, *VlMYB149* reduced ROS levels by enhancing antioxidant enzyme activity. Rather than weakening defences, it maintains cellular homeostasis to prevent oxidative damage, thereby enhancing disease resistance in a more precise and efficient manner. This mechanism is similar to that of certain *MYB* genes in *Arabidopsis* (Wang et al. 2016).

Typically, copper participate in Fenton‐like reactions, generating hydroxyl radicals and promoting ROS accumulation (Gaetke and Chow 2003). However, our findings demonstrate that *VlMYB149*‐induced copper enrichment was coupled with a significant upregulation of antioxidant enzymes (CAT, POD and SOD), effectively mitigating plant oxidative stress, which appears contradictory to the well‐established pro‐oxidant role of copper (Gaetke and Chow 2003). This is because copper can both promote the generation of ROS and simultaneously mitigate ROS‐induced oxidative damage by generating antioxidant enzymes (Pilon et al. 2006). Therefore, we propose that VlMYB149, by activating the expression of the membrane‐localised copper chaperone VlHIPP30, facilitates copper metabolism, potentially sequestering copper in a non‐redox‐active form or inducing it to generate antioxidant enzyme complexes. Moreover, the nuclear and membranous localisation of VlMYB149 implies a model whereby it perceives and transduces copper‐mediated signals at the membrane, ultimately leading to the direct regulation of transcriptional responses in the nucleus. This mechanism demonstrates that *VlMYB149* not only suppresses pathogen expansion via copper toxicity but also reinforces the cellular antioxidant capacity, highlighting a sophisticated strategy where copper homeostasis and ROS scavenging are synergistically optimised to confer resistance without incurring oxidative damage.

**FIGURE 8:**
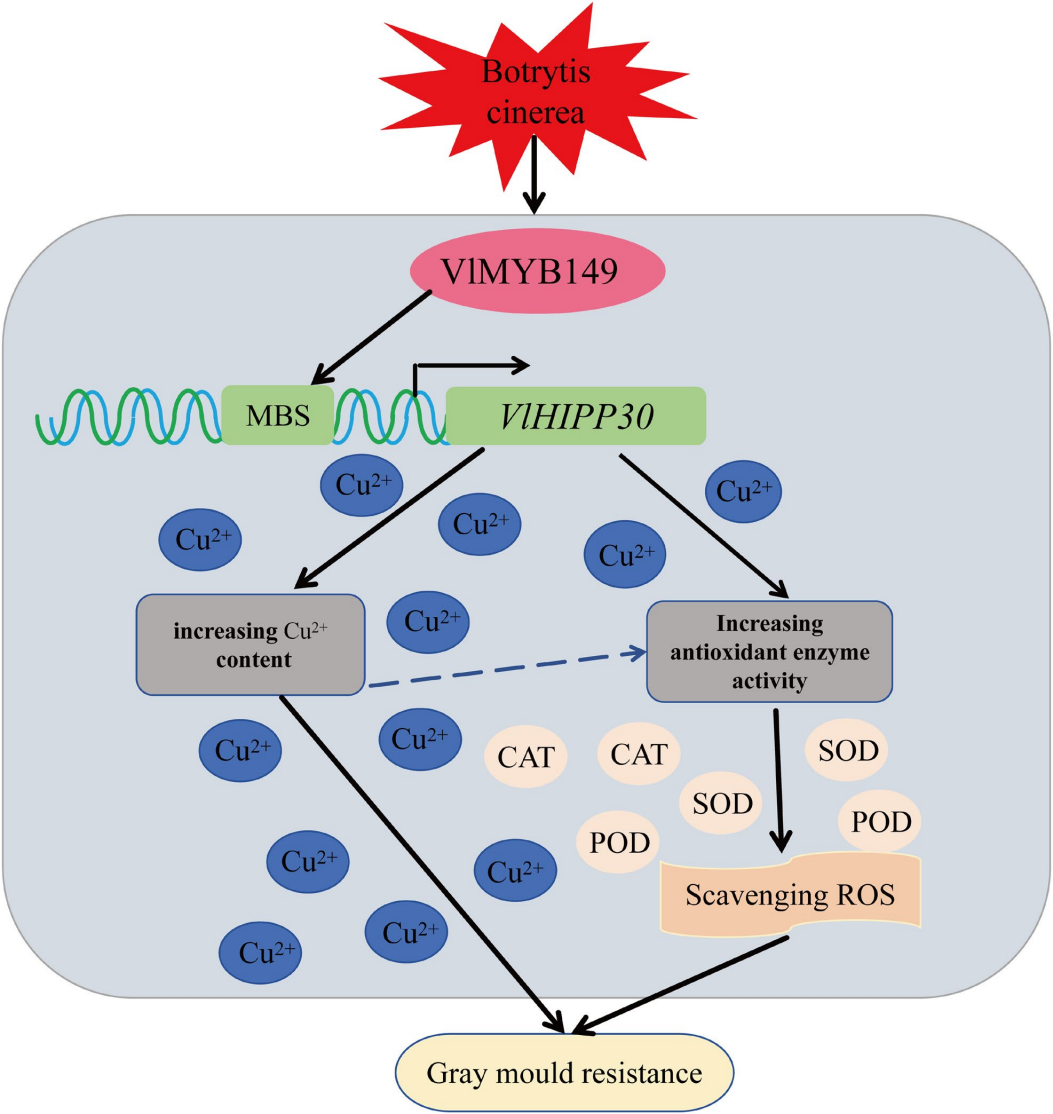
The proposed model displayed the mechanism of *VlMYB149* enhances the resistance to *Botrytis cinerea* in grapevine. When grape tissues are infected by *B. cinerea*, VlMYB149 is activated and binds to the MYB binding site (MBS) element in the promoter region of *VlHIPP30*, thereby promoting its expression. On one hand, VlMYB149 governs VlHIPP30 to facilitate copper ion accumulation in grape tissues, directly impeding pathogen invasion. On the other hand, the VlMYB149‐VlHIPP30 module collaboratively activates the antioxidant enzymes (catalase, CAT; POD, peroxidase; SOD, superoxide dismutase), which are responsible for scavenging the excessive and harmful reactive oxygen species (ROS) in grape tissues. Ultimately, the promotion of Cu^2+^ accumulation coupled with ROS scavenging enhances the resistance to grey mould in grapevine.

Thus, the VlMYB149‐VlHIPP30 module potentially boosts disease resistance via two pathways (Figure 8): *VlMYB149* governs *VlHIPP30* to facilitate copper accumulation at the cell membrane, directly impeding pathogen infiltration; the VlMYB149‐VlHIPP30 module collaboratively modulates antioxidant enzyme activity to eliminate ROS, which serve as signalling molecules for defence signal propagation. This mechanism not only enhances the regulatory roles of MYB TFs in grape disease resistance but also elucidates the function of *VlHIPP30* in this context. Furthermore, while this study elucidates the role of the VlMYB149‐VlHIPP30 module in enhancing disease resistance through dual mechanisms—copper accumulation and ROS scavenging—several key aspects remain to be fully elucidated. A primary limitation is that we have not yet fully deciphered how changes in copper content contribute to disease resistance, particularly the mechanistic basis of how exogenous copper application directly inhibits pathogen growth. The specific process through which copper exerts its antimicrobial effect—for instance, via disruption of microbial cell membranes, induction of metal toxicity or interference with essential enzymatic activities—warrants further investigation.

To address this, future studies should focus on the following directions: (1) Elucidating the copper‐mediated antimicrobial pathway. Detailed biochemical and cytological analyses are needed to examine the response of *B. cinerea* to copper facilitated by *VlHIPP30*. This may include assessing fungal membrane integrity, ROS dynamics in the pathogen, and the expression of fungal virulence genes under copper stress. Such experiments would clarify whether copper functions through membrane peroxidation, protein denaturation, or induction of metabolic toxicity. (2) Functional validation of PM‐localised defence initiation. As VlHIPP30 is membrane‐localised and contains an HMA domain, future work could investigate its role in PTI. For example, assessing whether VlHIPP30 participates in the formation of a recognition complex analogous to the HIPP1‐V/CMPG1‐V complex in wheat would strengthen our understanding of early defence signalling at the PM.

By addressing these questions, we can move closer to a comprehensive model wherein copper mobility and redox balance are coordinately regulated by the VlMYB149‐VlHIPP30 module, providing not only novel insights into grape disease resistance but also offering practical strategies for improving crop immunity through metal ion homeostasis and antioxidant engineering.

## 4 Experimental Procedures

### 4.1 Plant Materials and Growth Conditions

*Vitis labrusca* ‘Beta’, *V. vinifera* ‘Thompson Seedless’ and *V. vinifera* ‘Red Globe’ collected from the grape experimental garden at Shenyang Agricultural University, Shenyang, Liaoning, China (41°50′N, 123°24′E) were used as materials. *N. benthamiana* and *Arabidopsis* were grown in an artificial climate chamber at a controlled temperature of 25°C with a 16 h light photoperiod.

### 4.2 Pathogen Inoculation and Copper Treatments

The *B. cinerea* was isolated from RG and cultured on potato dextrose agar (PDA) for 2 weeks. *B. cinerea* was cultured at 22°C with 95% relative humidity. The spores were then collected using sterile water, and the spore suspension was adjusted to a concentration of 1 × 10^7^ spores/mL using a haemocytometer for inoculation. RG, TS and Beta leaves and fruits were separately infected with *B. cinerea* as previously described (Wang et al. 2015). After inoculation, all leaves and fruits were dark‐adapted for 24 h, followed by a 16‐h light photoperiod for 48 h. Then, samples were collected at 0, 24, 48, 72, 96 and 120 h for further analysis. The 0 h leaves infected with *B. cinerea* served as the control. Each treatment included three biological replicates, with each replicate including three grape leaves. Cu^2+^ treatment was carried out by spraying grape leaves with 200 μM CuCl_2_. Leaves were collected 0, 0.5, 1, 2, 6 and 10 h after treatment. The 0 h leaves sprayed with 200 μM CuCl_2_ solution served as the control. Each treatment included three biological replicates, with each replicate including three grape leaves. All samples were rapidly frozen in liquid nitrogen, then stored at −80°C for subsequent experiment.

### 4.3 RNA Extraction and Real‐Time Quantitative PCR Analysis

Total RNA was extracted using the RNA Mini Kit from Vazyme Biotechnology Co. Ltd. (Nanjing) according to the manual, and then reverse‐transcribed into cDNA using the 1st Strand cDNA Synthesis Kit from Vazyme Biotechnology Co. Ltd. following the instructions. The RT‐qPCR experiment was performed as described previously (Zhu, Zhang, et al. 2022; Zhu, Zhou, and Zhang 2022), with the *Actin* gene used as an internal reference to normalise the relative expression levels of genes. The stability of *Actin* gene expression across all experimental samples (including different grape varieties, *B. cinerea* inoculation time points, and treatment groups) was validated by semiquantitative RT‐PCR. Briefly, cDNA samples from each group were subjected to PCR amplification using *Actin*‐specific primers (listed in Table S1) under the following conditions: initial denaturation at 98°C for 5 min; followed by 32 cycles of denaturation at 94°C for 30 s, annealing at 60°C for 30 s, and extension at 72°C for 30 s; with a final extension at 72°C for 5 min. The PCR products were separated by 1.0% agarose gel electrophoresis and visualised by ethidium bromide staining. Consistent band intensities of *Actin* amplification products were observed across all samples, indicating stable expression of the *Actin* gene (Figure S3). The relative expression levels were calculated via the 2^−ΔΔC*t*^ method. The sequences of the primers used in this study are listed in the Table S1.

### 4.4 Bioinformatics Analysis of Grape *MYB149*

The chromosomal localisation of the *MYB149* was determined using the NCBI database (https://www.ncbi.nlm.nih.gov/). The coding and promoter sequences of grape *MYB149* were obtained from grape cultivar Beta leaves. The conserved domains of the MYB149 protein were analysed using the SMART website (https://smart.embl.de/). Amino acid sequences of MYB family members from grape and *Arabidopsis* were retrieved from the NCBI database. The phylogenetic trees were constructed using the neighbour‐joining method with 1200 bootstrap replicates in MEGA 11 software.

### 4.5 Subcellular Localisation

The coding sequences of *VlMYB149* and *VlHIPP30* were individually inserted into pCAMBIA2300‐eGFP, resulting in VlMYB149‐eGFP and VlHIPP30‐eGFP. Using the EV as a control, the fusion vectors were co‐transformed with the nuclear marker (H2B‐mCherry) into 6‐week‐old *N. benthamiana* leaves. After 1 day of culture in the dark followed by 2 days of culture under a normal light cycle, protein localisation was observed using a Zeiss LSM980 microscope combined with H2B‐mCherry.

### 4.6 Gene Transformation in Grape and *B. cinerea* Inoculation

The coding sequences of *VlMYB149* and *VlHIPP30* were individually inserted into pCAMBIA2300, forming OE‐*VlMYB149* and OE‐*VlHIPP30*. To silence *MYB149* expression in grape plants, a target fragment (300 bp) of the coding region was inserted into the RNAi vector (pFGC5941) to create the *VlMYB149* silencing vector RNAi‐*VlMYB149*. The method for transient transformation in grape leaves was performed as previously described (Jiang et al. 2021), and leaves were treated with *B. cinerea* using the agar disk inoculation method (Zhu, Zhang, et al. 2022; Zhu, Zhou, and Zhang 2022). The original *B. cinerea* strain was cultured on PDA in the dark at 22°C for 2 weeks. Agar disks (0.5 cm in diameter) with uniformly distributed *B. cinerea* mycelia were prepared using a puncher, and the mycelium‐containing side was pressed onto grape leaves. Inoculated leaves were placed in trays under high humidity (> 90%), incubated in the dark at 22°C for 24 h, and then cultured under a normal light cycle. Leaves transformed with the EV were inoculated as controls in the same manner. Leaves were collected at 24, 72 and 120 hpi for phenotypic observation and *B. cinerea* biomass quantification, with the same leaves used for both quantitative analysis and phenotypic observation. Each treatment included three biological replicates, with every replicate including at least eight leaves.

### 4.7 Gene Stable Overexpression in *Arabidopsis* and *B. cinerea* Inoculation

Genetic transformation in *Arabidopsis* was performed as previously described by Zhu, Zhang, et al. (2022) and Zhu, Zhou, and Zhang (2022). The screening process of *VlMYB149*‐overexpressing *Arabidopsis* plants is shown in Figure S4. *B. cinerea* was inoculated on PDA and cultured in a 22°C incubator for 2 weeks for later use. *Arabidopsis* plants were inoculated with a *B. cinerea* spore suspension at a concentration of 1 × 10^7^ spores/mL. All infected plants were cultured in an incubator at 22°C.

### 4.8 Determination of Physiological Indexes Related to Plant Disease Resistance

The biomass of *B. cinerea* in transgenic grape and wild‐type lines at different inoculation time points was analysed using quantitative real‐time PCR (qPCR) (Zhu, Zhang, et al. 2022; Zhu, Zhou, and Zhang 2022). The relative biomass was calculated as qRT‐*Bc*Actin/AtActin2 or qRT‐*Bc*Actin/Actin7 using primers for *B. cinerea* *Actin* (qRT‐*Bc*Actin) listed in Table S1. *B. cinerea* biomass was determined in three biological replicates (primers are listed in Table S1). To observe cell death, 24, 72, 120 hpi leaves were submerged in trypan blue solution (20 mL ethanol, 10 mL phenol, 10 mL lactic acid and 10 mg trypan blue dissolved in 10 mL sterile water) and boiled for 2 min. The stained leaves were bleached with 2.5 g/mL chloral hydrate solution. Leaves were collected 24, 72, 120 hpi for DAB staining and NBT staining by immersion in a 1 mg/mL DAB solution (pH 3.8) or DAB solution for 24 h and then boiled in 95% ethanol for destaining. The measurement of electrolyte leakage rate is based on an existing method and slightly modified (Zhu, Zhang, et al. 2022; Zhu, Zhou, and Zhang 2022). Ten leaf discs (0.5 cm diameter) from five leaves were incubated in 15 mL centrifuge tube with 5 mL of double‐distilled water. After shaking (100 rpm, 25°C, 20 min), the initial conductivity (C_1_) was recorded using FE30 (Mettler Toledo Instruments Co. Ltd.). Samples in centrifuge tubes were then boiled for 20 min, followed by shaking again at 25°C, and the second conductivity (C_2_) was measured. The electrolyte leakage (EL) was calculated as: EL (%) = (C_1_/C_2_) × 100. The activities of CAT, SOD and POD were assayed using commercial kits from Keming Biotechnology Co. Ltd. The contents of H_2_O_2_ and MDA, as well as copper concentration, were determined using kits from Soleibao Biotechnology Co. Ltd., following the respective instructions. Each treatment included three biological replicates.

The *B. cinerea* biomass in *Arabidopsis* was determined in three biological replicates (primers are listed in Table S1). After 3 days of inoculation, leaves were collected for DAB and NBT staining by immersion in a 1 mg/mL DAB solution (pH 3.8) or NBT solution for 24 h and then boiled in 95% ethanol for destaining. The contents of H_2_O_2_, MDA and copper, as well as the activities of CAT, POD and SOD, were measured in *Arabidopsis* leaves were tested using detection kit from Soleibao Biotechnology Co. Ltd. following the instructions.

### 4.9 Yeast One‐Hybrid Assay

The coding sequence of *VlMYB149* was inserted into the pGADT7 vector to construct AD‐VlMYB149. The promoter sequences of *VlHIPP30* were cloned into the pAbAi vector to construct pAbAi‐VlHIPP30. The yeast strains carrying these fusion vectors and EV were cultured on SD/−Leu medium supplemented with AbA to validate protein–DNA interaction.

### 4.10 Dual‐Luciferase Reporter Assay

The coding sequence of *VlMYB149* was inserted into pCAMBIA2300 vector to construct the effector. The 2 kb promoter sequence of *VlHIPP30* was inserted into pGreenII0800‐LUC vector to form the reporter. Then, the recombinant vectors were co‐transformed into *N. benthamiana* leaves via *A. tumefaciens* GV3101. LUC signals were observed using an vivo fluorescence imager from Tanon Biotechnology Co. Ltd., and LUC/REN activity was measured using luciferase detection kit from Biyuntian Biotechnology Co. Ltd., following the instructions. Each treatment included three biological replicates, with three technical replicates per biological replicate.

### 4.11 GUS Reporter Assay

The promoter sequence of *VlHIPP30* was inserted into pC0390‐35S‐GUS vector to construct the reporter. The fusion vector and effector (*VlMYB149*) were co‐transformed into *N. benthamiana* leaves via *A. tumefaciens* GV3101. The pC0390‐35S‐GUS (CaMV35S‐GUS) and pC0390‐GUS vector was used as positive control and negative control, respectively. GUS staining was performed using the GUS staining agent, and GUS activity was determined using the GUS enzyme detection kit from Biyuntian Biotechnology Co. Ltd., following the instructions. Each treatment included three biological replicates, with three technical replicates per biological replicate.

### 4.12 Statistical Analysis

The differences between two groups were assessed by Student's *t* test, with statistical significance set at *p* < 0.05. For multiple comparisons, one‐way analysis of variance (ANOVA) followed by Tukey's multiple comparison test was used, and significance was determined at *p* < 0.05. All statistical analyses were performed using GraphPad Prism v. 8.0 software.

## Author Contributions

Xiaoli Zhang performed most experiments, wrote and modified the original draft. Xiangyu Zhou supplemented experimental results. Yanqiu Du and Yuling Li performed the data curation. Hong Lin and Feng Sun supervised and reviewed the research plans. Yong Wang and Songlin Zhou analysed the data. Guo Yinshan, Jiang Changyue and Guohong Wu designed the research and modified the manuscript. All authors read and approved the final manuscript.

## Funding

This work was supported by the National Natural Science Foundation of China (Grant 32402494), the China Agriculture Research System (Grant CARS‐29‐yc‐6), the Major Agricultural Science Projects of Liaoning Province (Grant 2023JH1/10200004), the Natural Science Foundation of Liaoning Province (grant no. 2023‐BSBA‐278), the Project of Education Department of Liaoning Province (Grant JYTYB2024030), the Tianchi Talent Program Project in Xinjiang Uygur Autonomous Region (Grant 2023‐02‐06) and the Science and Technology Program of Shenyang (Grant 23‐410‐2‐03).

## Conflicts of Interest

The authors declare no conflicts of interest.

## Supporting information


**Table S1:** The primers used in the study.

**Figure S1:** Analysis of the expression patterns of MYB family members under *Bc* inoculation in ‘Beta’ and ‘RG’ grapevines on the basis of RNA‐seq.

**Figure S2:** Analysis of the expression characteristics of MYBs (MYB1, MYB3, MYB53, MYB57, MYB68, MYB77, MYB82, MYB149) under *Bc* inoculation in different grape varieties.

**Figure S3:** The Actin gene semi‐quantitative RT‐PCR analysis. A: Expression of the Actin gene in various grape varieties under *Bc* infection; B: Expression of the Actin gene in different tissues among various varieties. a, stem; b, young stem; c, young leaf; d, mature leaf; e, older leaf; f, tendril; g, flower head; h, mature fruit.

**Figure S4:** The screening and identification of stable *VlMYB149‐*overexpressing *Arabidopsis*. (A–C) The screening process of *VlMYB149*‐overexpressing *Arabidopsis*; (D–E) The molecular identification of *VlMYB149* in transgenic *Arabidopsis*.

## Data Availability

All relevant data and charts of this study can be found in the article and Supporting Information.
