# Supplementary material for: The VlMYB149‐VlHIPP30 Regulatory Module Enhances Grapevine Resistance to Botrytis cinerea by Activating the Antioxidant System and Copper Metabolism
Source: Mol Plant Pathol. 2026 Jan 11;27(1):e70197. doi: 10.1111/mpp.70197 (PMC12791032; doi:10.1111/mpp.70197)
Supplement: Supplementary file 2 — Figure S1: Analysis of the expression patterns of MYB family members under Bc inoculation in ‘Beta’ and ‘RG’ grapevines on the basis of RNA‐seq. [file MPP-27-e70197-s004.docx]

**Supplementary Figure 1. Analysis of the expression patterns of MYB family members under *Bc* inoculation in ‘Beta’ and ‘RG’ grapevines on the basis of RNA-seq.**
