# Supplementary material for: The VlMYB149‐VlHIPP30 Regulatory Module Enhances Grapevine Resistance to Botrytis cinerea by Activating the Antioxidant System and Copper Metabolism
Source: Mol Plant Pathol. 2026 Jan 11;27(1):e70197. doi: 10.1111/mpp.70197 (PMC12791032; doi:10.1111/mpp.70197)
Supplement: Supplementary file 3 — Figure S2: Analysis of the expression characteristics of MYBs (MYB1, MYB3, MYB53, MYB57, MYB68, MYB77, MYB82, MYB149) under Bc inoculation in different grape varieties. [file MPP-27-e70197-s002.docx]

**Supplementary Figure 2. Analysis of the expression characteristics of MYBs (MYB1, MYB3, MYB53, MYB57, MYB68, MYB77, MYB82, MYB149) under *Bc* inoculation in different grape varieties.**
