# Supplementary material for: The VlMYB149‐VlHIPP30 Regulatory Module Enhances Grapevine Resistance to Botrytis cinerea by Activating the Antioxidant System and Copper Metabolism
Source: Mol Plant Pathol. 2026 Jan 11;27(1):e70197. doi: 10.1111/mpp.70197 (PMC12791032; doi:10.1111/mpp.70197)
Supplement: Supplementary file 4 — Figure S3: The Actin gene semi‐quantitative RT‐PCR analysis. A: Expression of the Actin gene in various grape varieties under Bc infection; B: Expression of the Actin gene in different tissues among various varieties. a, stem; b, young stem; c, young leaf; d, mature leaf; e, older leaf; f, tendril; g, flower head; h, mature fruit. [file MPP-27-e70197-s001.docx]

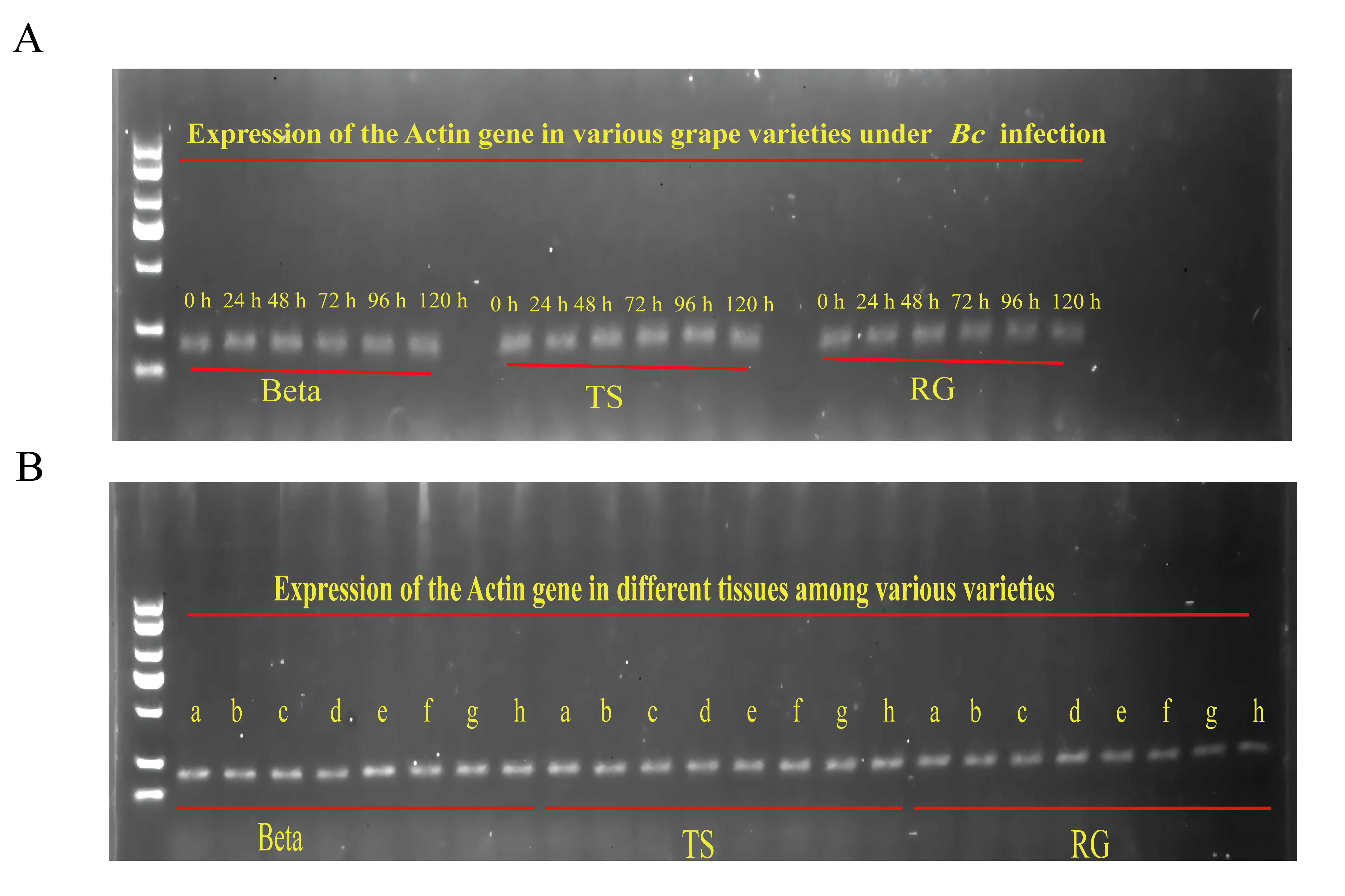


**Supplementary Figure 3. The Actin gene semi-quantitative** **RT-PCR analysis.** A: Expression of the Actin gene in various grape varieties under *Bc* infection; B: Expression of the Actin gene in different tissues among various varieties. a, stem; b, young stem; c, young leaf; d, mature leaf; e, older leaf; f, tendril; g, flower head; h, mature fruit.
