# Supplementary material for: The VlMYB149‐VlHIPP30 Regulatory Module Enhances Grapevine Resistance to Botrytis cinerea by Activating the Antioxidant System and Copper Metabolism
Source: Mol Plant Pathol. 2026 Jan 11;27(1):e70197. doi: 10.1111/mpp.70197 (PMC12791032; doi:10.1111/mpp.70197)
Supplement: Supplementary file 5 — Figure S4: The screening and identification of stable VlMYB149‐overexpressing Arabidopsis. (A–C) The screening process of VlMYB149‐overexpressing Arabidopsis; (D–E) The molecular identification of VlMYB149 in transgenic Arabidopsis. [file MPP-27-e70197-s005.docx]

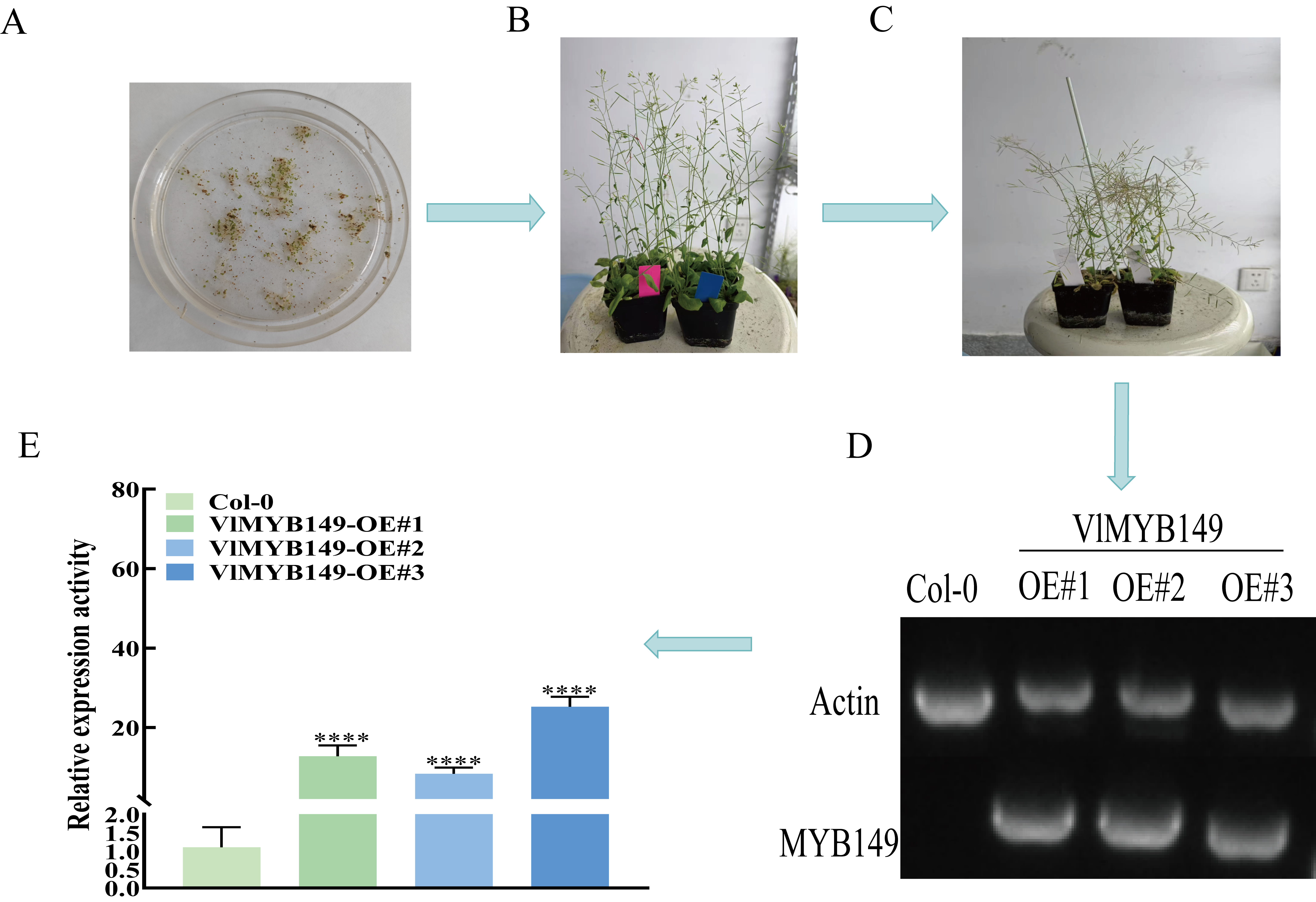


**Supplementary Figure 4. The screening and identification of stable *VlMYB149-*overexpressing *Arabidopsis.***

A-C: The screening process of *VlMYB149*-overexpressing *Arabidopsis*; D-E: The molecular identification of *VlMYB149* in transgenic *Arabidopsis.*
